# Clay minerals and major elements concentrations of Zhuanglang Miocene red clay in Longzhong Basin, China

**DOI:** 10.1016/j.dib.2018.01.030

**Published:** 2018-01-31

**Authors:** Qiansuo Wang, Yougui Song

**Affiliations:** a Shandong Provincial Key Laboratory of Water and Soil Conservation and Environmental Protection, College of Resources and Environment, Linyi University, Linyi 276000, China; b State Key Laboratory of Loess and Quaternary Geology, Institute of Earth Environment, Chinese Academy of Sciences, Xi'an 710061, China

## Abstract

This article presents clay minerals and major elements data of Miocene red clay from Zhuanglang core (ZL) from the eastern Longzhong Basin, China. The dataset including the contents of main clay minerals such as smectite, kaolinite, illite and chlorite and other important clay minerals parameters as illite crystallinity, Illite 5 Å/10 Å, kaolinite/(illite +chlorite) ratio and major elements and its ratio as SiO_2_, Al_2_O_3_, MgO, Na_2_O, K_2_O, K_2_O/Al_2_O_3_ and SiO_2_/Al_2_O_3_ in the Mid-Miocene climatic optimum (MMCO). The X-ray diffraction (XRD) and X-ray Fluorescence (XRF) experiments were made at the State Key Laboratory of Loess and Quaternary Geology, Institute of Earth Environment, Chinese Academy of Sciences. The data provide the evidence for understanding the MMCO and its driving factors.

**Specifications Table**

**Table:** 

Subject area	Earth science
More specific subject area	Paleoclimate
Type of data	Table, figure
How data was acquired	Field survey, sample collection and laboratory analysis
Data format	Raw data collection and Analysis
Experimental factors	*Samples dry completely in room temperature before measuring*
Experimental features	The air-dry samples were treated with H_2_O_2_ to remove organic matter and dilute HCl to remove the calcium carbonate; the clay fractions were separated by gravity separation using Stoke's Law. X-ray diffraction (XRD) analysis of the oriented mounts was carried out for each sample under natural (air-dried) conditions (N), ethylene-glycol solvation for 24 h in a desiccator (EG), and heating at 550 °C for 2 h (H). XRD patterns were obtained using a PANalytical X'Pert Pro MPD diffractometer with CuKα radiation and Ni filter, under a voltage of 40 kV and a current of 40 mA. The scans were performed from 3° to 30°, with a step size of 0.0167°. And a high resolution scan from 24° to 26° 2θ at a rate of 0.1° 2θ per minute was carried out to isolate the contributions of kaolinite and chlorite in the 3.5 Å peak. The major elements of clay fractions were determined using a PANalytical PW4400 X-ray fluorescence (XRF) spectrometer.
Data source location	35°13′N, 106°05′E, Zhuanglang drilling core, Zhuanglang County, Gansu Province, China
Data accessibility	*Data are within this article and related references*
Related research article	Song et al. [1]

**Value of the data**

• Provide basic clay mineral data for both local and global comparisons.
• Data could be used to display the contents of main clay minerals and its paleoclimatic characteristics in the MMCO period.
• Data provided a reasonable interpretation for the MMCO event.
• Data given here could motivate the studies on clay minerals in future.

## 1 Data

**Fig. 1:**
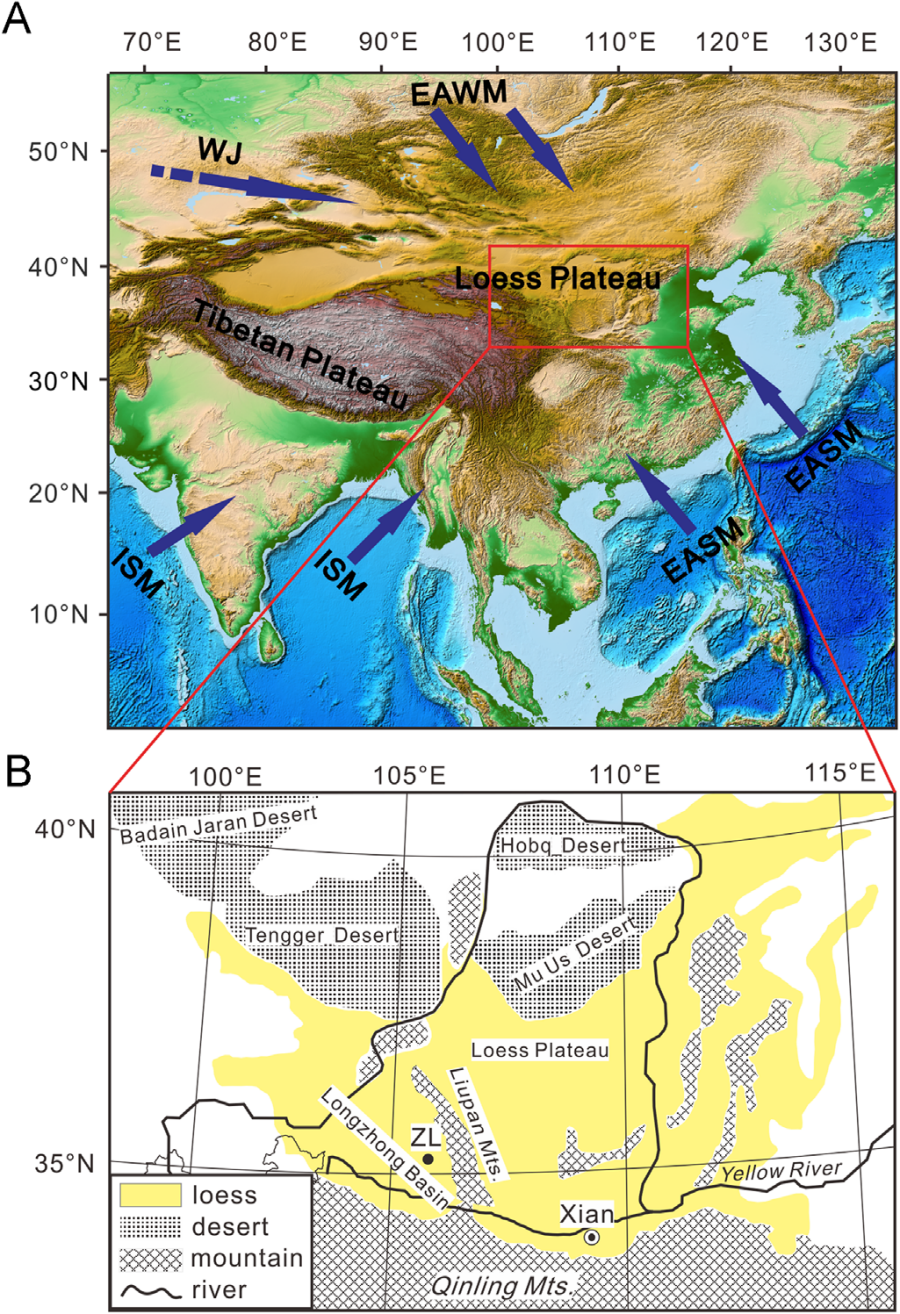
Map showing (A) the location of the ZL drill core (black spot) on the Chinese Loess Plateau and (B) the atmospheric circulation system of China. EASM: East Asian Summer Monsoon; EAWM: East Asian Winter Monsoon; ISM: Indian Summer Monsoon; WJ: Westerly Jet. Maps (A) was created by ETOPO1 (doi:10.7289/V5C8276M, https://www.ngdc.noaa.gov/mgg/global/global.html).

This article presents clay minerals and major elements data of Miocene red clay from Zhuanglang (ZL) core from the eastern Longzhong Basin, China (Fig. 1). The concentrations of main clay minerals includes mectite, kaolinite, illite and chlorite and other important clay minerals parameters as illite crystallinity, kaolinite/(illite + chlorite) ratio. The main clay minerals types, contents, illite crystallinity, illite 5 Å/10 Å, kaolinite/(illite+chlorite) from 17 to 12 Ma are given in Table 1. The major elements include SiO_2_, Al_2_O_3_, MgO, Na_2_O, K_2_O. The major elements of clay fractions and their ratios from 17 to 12 Ma of the ZL red clay are showed in Table 2.

**Table 1:** Clay minerals contents and characteristic indexes of the ZL red clay from 17 to 12 Ma.

**Sample ID**	**Depth**	**Age**	**Smectite**	**Kaolinite**	**Illite**	**Chlorite**	**Illite crystallinity**	**Illite 5** **Å/10** **Å**	**K/(I+Ch)**
	**(m)**	**(Ma)**	**(%)**	**(%)**	**(%)**	**(%)**			
ZL240	240	11.93	14.62	13.25	57.95	14.18	0.47	0.67	0.18
ZL242	242	12.02	16.33	10.89	58.75	14.02	0.44	0.70	0.15
ZL244	244	12.10	10.75	11.09	64.21	13.95	0.41	0.67	0.14
ZL246	246	12.18	5.58	12.35	65.23	16.84	0.45	0.70	0.15
ZL248	248	12.26	5.24	11.34	67.55	15.88	0.44	0.71	0.14
ZL250	250	12.34	4.54	11.17	67.77	16.52	0.41	0.70	0.13
ZL252	252	12.42	5.95	11.58	65.51	16.96	0.46	0.70	0.14
ZL254	254	12.49	7.34	10.81	67.08	14.77	0.40	0.71	0.13
ZL256	256	12.55	7.36	11.47	64.30	16.86	0.45	0.70	0.14
ZL258	258	12.62	11.73	10.21	64.36	13.71	0.43	0.66	0.13
ZL260	260	12.72	5.36	10.25	67.71	16.68	0.45	0.66	0.12
ZL262	262	12.76	7.69	9.68	69.76	12.86	0.44	0.65	0.12
ZL264	264	12.82	5.52	9.98	71.13	13.37	0.40	0.66	0.12
ZL266	266	12.89	4.74	9.41	73.35	12.50	0.40	0.70	0.11
ZL268	268	12.96	5.70	11.07	66.51	16.72	0.41	0.67	0.13
ZL270	270	13.02	9.53	12.56	68.23	12.48	0.45	0.69	0.16
ZL272	272	13.07	9.55	12.57	69.24	11.99	0.46	0.67	0.15
ZL274	274	13.13	8.66	11.45	69.10	11.38	0.57	0.70	0.14
ZL276	276	13.19	7.34	12.20	69.09	11.37	0.45	0.69	0.15
ZL278	278	13.25	8.14	10.74	69.70	11.43	0.48	0.71	0.13
ZL280	280	13.31	9.53	11.68	68.39	10.41	0.46	0.71	0.15
ZL282	282	13.37	8.81	10.92	69.13	11.14	0.41	0.73	0.14
ZL284	284	13.42	9.19	11.45	68.77	10.59	0.42	0.71	0.14
ZL286	286	13.48	8.74	10.92	69.07	11.27	0.45	0.66	0.14
ZL288	288	13.53	8.35	10.58	69.52	11.54	0.42	0.65	0.13
ZL290	290	13.58	8.65	10.91	69.31	11.12	0.44	0.70	0.14
ZL292	292	13.62	8.05	10.17	70.15	11.63	0.40	0.68	0.12
ZL294	294	13.67	7.49	9.40	70.84	12.27	0.42	0.66	0.11
ZL296	296	13.71	8.53	10.14	69.42	11.25	0.45	0.68	0.13
ZL298	298	13.74	8.72	11.37	68.84	10.70	0.45	0.71	0.14
ZL300	300	13.80	9.14	10.87	70.13	11.14	0.44	0.70	0.13
ZL302	302	13.84	7.94	11.48	70.11	11.54	0.43	0.68	0.14
ZL304	304	13.89	9.25	10.81	69.47	12.26	0.44	0.72	0.13
ZL306	306	13.93	7.66	10.57	68.99	12.34	0.44	0.66	0.13
ZL308	308	13.98	8.33	10.39	69.60	12.45	0.44	0.67	0.13
ZL310	310	14.02	10.63	11.58	69.22	11.37	0.46	0.51	0.14
ZL312	312	14.06	10.72	11.96	64.60	10.93	0.47	0.46	0.16
ZL314	314	14.11	12.46	11.66	63.30	9.07	0.48	0.47	0.16
ZL316	316	14.16	11.86	13.73	64.65	9.76	0.46	0.52	0.18
ZL318	318	14.20	12.30	12.03	63.94	10.22	0.47	0.48	0.16
ZL320	320	14.25	19.83	13.04	54.58	9.54	0.47	0.52	0.20
ZL322	322	14.29	22.70	10.78	59.01	7.51	0.47	0.52	0.16
ZL324	324	14.33	26.77	12.87	59.92	5.45	0.49	0.56	0.20
ZL326	326	14.38	25.95	12.47	51.50	6.58	0.52	0.50	0.21
ZL328	328	14.42	27.88	11.57	52.66	6.29	0.49	0.49	0.20
ZL330	330	14.47	23.20	12.39	53.44	7.87	0.44	0.50	0.20
ZL332	332	14.51	22.96	11.93	52.09	7.32	0.48	0.47	0.20
ZL334	334	14.55	26.25	11.70	49.04	8.02	0.47	0.50	0.21
ZL336	336	14.60	17.90	10.32	54.66	7.82	0.47	0.49	0.17
ZL338	338	14.65	21.01	11.61	60.09	7.48	0.47	0.54	0.17
ZL340	340	14.69	20.54	12.99	57.89	9.57	0.47	0.43	0.19
ZL342	342	14.74	17.68	12.77	58.95	8.00	0.48	0.47	0.19
ZL344	344	14.79	19.45	10.29	56.80	8.08	0.48	0.46	0.16
ZL346	346	14.85	18.14	11.14	62.47	8.25	0.68	0.45	0.16
ZL348	348	14.91	19.27	13.23	60.58	10.92	0.51	0.52	0.19
ZL350	350	14.96	20.60	10.91	55.67	9.72	0.44	0.50	0.17
ZL352	352	15.04	22.38	14.61	57.51	9.49	0.53	0.48	0.22
ZL354	354	15.11	18.85	13.71	57.42	9.33	0.48	0.50	0.21
ZL356	356	15.18	19.26	13.57	55.24	8.83	0.49	0.48	0.21
ZL358	358	15.26	21.72	12.92	56.82	8.34	0.59	0.49	0.20
ZL360	360	15.34	17.28	13.02	61.26	8.54	0.49	0.50	0.19
ZL362	362	15.42	19.54	10.73	57.53	9.20	0.49	0.50	0.16
ZL364	364	15.49	20.53	11.76	54.32	9.39	0.50	0.55	0.18
ZL366	366	15.58	21.15	12.13	57.78	8.94	0.60	0.47	0.18
ZL368	368	15.64	18.57	13.78	55.43	8.98	0.56	0.48	0.21
ZL370	370	15.74	15.66	14.23	61.99	9.12	0.47	0.49	0.20
ZL372	372	15.82	18.55	12.03	60.13	9.29	0.48	0.48	0.17
ZL374	374	15.90	14.37	13.91	56.20	9.51	0.66	0.52	0.21
ZL376	376	15.98	17.10	12.41	60.35	9.14	0.64	0.48	0.18
ZL378	378	16.08	8.53	8.86	67.28	15.34	0.41	0.58	0.11
ZL380	380	16.18	2.69	7.57	75.92	13.82	0.39	0.61	0.08
ZL382	382	16.28	2.98	7.55	74.72	14.75	0.39	0.64	0.08
ZL384	384	16.32	5.80	9.28	71.72	13.19	0.46	0.69	0.11
ZL386	386	16.40	3.39	8.58	73.45	14.57	0.41	0.71	0.10
ZL388	388	16.52	3.26	8.12	73.85	14.77	0.45	0.71	0.09
ZL390	390	16.70	8.62	9.02	67.98	14.38	0.43	0.72	0.11
ZL392	392	16.80	3.99	8.36	71.77	15.88	0.43	0.69	0.10
ZL394	394	16.88	5.71	8.73	71.46	14.10	0.46	0.69	0.10
ZL396	396	16.96	7.85	8.28	70.67	13.20	0.44	0.72	0.10
ZL398	398	17.04	2.30	6.60	73.77	17.33	0.40	0.69	0.07

**Table 2:** The contents of major elements of clay fractions of the ZL red clay from 17 to 12 Ma.

**Sample ID**	**Depth (m)**	**Age (Ma)**	**SiO**_**2**_	**Al**_**2**_**O**_**3**_	**MgO**	**Na**_**2**_**O**	**K**_**2**_**O**	**K**_**2**_**O/Al**_**2**_**O**_**3**_	**SiO**_**2**_**/Al**_**2**_**O**_**3**_
ZL242	242	12.02	47.62	20.53	4.16	0.74	3.83	0.19	2.32
ZL244	244	12.10	47.98	20.66	4.26	0.75	3.99	0.19	2.32
ZL246	246	12.18	48.21	20.53	4.38	0.78	4.03	0.20	2.35
ZL248	248	12.26	48.03	20.65	4.33	0.77	4.17	0.20	2.33
ZL250	250	12.34	48.68	20.75	4.27	0.80	4.22	0.20	2.35
ZL252	252	12.42	48.09	20.68	4.31	0.78	4.18	0.20	2.33
ZL254	254	12.49	48.98	20.93	4.17	0.79	4.20	0.20	2.34
ZL256	256	12.55	47.93	20.93	4.42	0.80	4.23	0.20	2.29
ZL258	258	12.62	48.77	20.93	4.13	0.74	4.00	0.19	2.33
ZL260	260	12.72	47.87	20.87	4.17	0.77	4.10	0.20	2.29
ZL262	262	12.76	48.22	20.94	4.13	0.77	4.11	0.20	2.30
ZL264	264	12.82	47.71	20.91	3.97	0.77	4.14	0.20	2.28
ZL266	266	12.89	48.27	20.84	4.08	0.76	4.23	0.21	2.32
ZL268	268	12.96	47.86	20.55	4.06	0.71	4.12	0.20	2.33
ZL270	270	13.02	48.14	20.50	4.04	0.71	4.31	0.21	2.35
ZL272	272	13.07	48.38	20.79	4.06	0.70	4.05	0.20	2.33
ZL274	274	13.13	48.22	20.92	4.11	0.71	3.86	0.19	2.30
ZL276	276	13.19	48.43	20.78	4.46	0.73	3.82	0.19	2.33
ZL278	278	13.25	48.52	20.71	4.25	0.72	3.89	0.19	2.34
ZL280	280	13.31	47.99	20.53	3.92	0.71	3.75	0.19	2.34
ZL282	282	13.37	48.39	21.04	3.57	0.69	3.75	0.18	2.30
ZL284	284	13.42	48.46	21.05	3.86	0.72	3.87	0.19	2.30
ZL286	286	13.48	47.57	20.84	3.86	0.67	3.76	0.18	2.28
ZL288	288	13.53	48.27	20.70	3.85	0.69	3.74	0.18	2.33
ZL290	290	13.58	47.45	20.62	3.96	0.72	3.78	0.18	2.30
ZL292	292	13.62	47.91	21.09	3.97	0.68	3.78	0.18	2.27
ZL294	294	13.67	47.76	20.59	4.03	0.68	3.81	0.18	2.32
ZL296	296	13.71	48.79	21.02	4.38	0.79	3.92	0.19	2.32
ZL298	298	13.74	48.81	20.88	4.05	0.74	4.10	0.19	2.34
ZL300	300	13.80	47.91	20.63	3.81	0.70	3.98	0.19	2.32
ZL302	302	13.84	48.13	20.61	3.95	0.70	3.95	0.19	2.34
ZL304	304	13.89	48.45	20.71	3.66	0.71	3.91	0.19	2.34
ZL306	306	13.93	47.66	20.52	3.83	0.70	4.00	0.19	2.32
ZL308	308	13.98	48.28	20.82	4.01	0.74	3.91	0.19	2.32
ZL310	310	14.02	47.78	20.91	3.99	0.76	3.99	0.19	2.29
ZL312	312	14.06	47.59	21.22	3.77	0.73	4.00	0.19	2.24
ZL314	314	14.11	47.31	21.36	3.91	0.75	3.81	0.18	2.21
ZL316	316	14.16	47.64	21.43	3.88	0.70	3.89	0.19	2.22
ZL318	318	14.20	46.31	20.87	4.16	0.76	3.73	0.18	2.22
ZL320	320	14.25	47.46	20.64	3.91	0.80	3.61	0.17	2.30
ZL322	322	14.29	47.21	20.88	4.09	0.77	4.10	0.20	2.26
ZL324	324	14.33	46.87	21.12	4.68	0.76	4.11	0.20	2.22
ZL326	326	14.38	46.52	20.61	4.21	0.83	3.82	0.19	2.26
ZL328	328	14.42	46.23	21.22	4.06	0.73	3.70	0.18	2.18
ZL330	330	14.47	46.91	21.76	3.87	0.74	4.13	0.20	2.16
ZL332	332	14.51	47.18	20.98	4.09	0.77	3.92	0.19	2.25
ZL334	334	14.55	47.64	20.81	4.36	0.79	3.85	0.19	2.29
ZL336	336	14.60	46.24	20.44	4.35	0.79	3.72	0.19	2.26
ZL338	338	14.65	46.63	21.06	4.14	0.73	3.83	0.19	2.21
ZL340	340	14.69	47.05	20.84	4.02	0.71	3.89	0.19	2.26
ZL342	342	14.74	46.49	20.91	4.35	0.74	3.77	0.18	2.22
ZL344	344	14.79	46.55	20.62	3.95	0.76	3.85	0.19	2.26
ZL346	346	14.85	47.24	21.22	4.06	0.69	3.85	0.19	2.23
ZL348	348	14.91	47.15	21.31	4.21	0.78	3.85	0.19	2.21
ZL350	350	14.96	46.80	20.98	4.15	0.72	4.32	0.20	2.23
ZL352	352	15.04	47.14	21.31	4.51	0.74	4.19	0.20	2.21
ZL354	354	15.11	47.34	21.43	3.86	0.75	4.19	0.19	2.21
ZL356	356	15.18	46.55	20.85	4.18	0.75	4.02	0.19	2.23
ZL358	358	15.26	46.21	20.96	4.16	0.70	4.14	0.20	2.20
ZL360	360	15.34	45.99	21.02	4.25	0.74	3.90	0.19	2.19
ZL362	362	15.42	45.84	20.86	4.05	0.77	3.92	0.19	2.20
ZL364	364	15.49	46.26	20.86	4.08	0.75	4.11	0.19	2.22
ZL366	366	15.58	46.18	20.85	4.23	0.75	4.23	0.20	2.21
ZL368	368	15.64	47.01	21.40	4.06	0.80	3.99	0.19	2.20
ZL370	370	15.74	46.66	21.03	4.05	0.73	4.18	0.20	2.22
ZL372	372	15.82	47.35	21.13	3.95	0.74	4.09	0.19	2.24
ZL374	374	15.90	46.84	21.01	4.03	0.77	3.95	0.19	2.23
ZL376	376	15.98	47.22	21.22	4.29	0.75	3.81	0.18	2.23
ZL378	378	16.08	47.93	20.53	3.91	0.80	4.05	0.19	2.33
ZL380	380	16.18	48.36	20.69	4.08	0.72	4.46	0.21	2.34
ZL382	382	16.28	47.58	20.55	4.15	0.72	4.41	0.21	2.32
ZL384	384	16.32	47.81	20.61	4.18	0.74	4.13	0.20	2.32
ZL386	386	16.40	47.50	20.66	4.19	0.74	4.36	0.21	2.30
ZL388	388	16.52	47.29	20.27	4.38	0.74	4.22	0.20	2.33
ZL390	390	16.70	48.01	20.73	4.32	0.75	3.98	0.19	2.32
ZL392	392	16.80	47.34	20.47	4.16	0.69	4.29	0.20	2.31
ZL394	394	16.88	47.92	20.66	4.33	0.76	3.99	0.19	2.32
ZL396	396	16.96	48.86	21.15	4.29	0.74	3.91	0.19	2.31

**Fig. 2:**
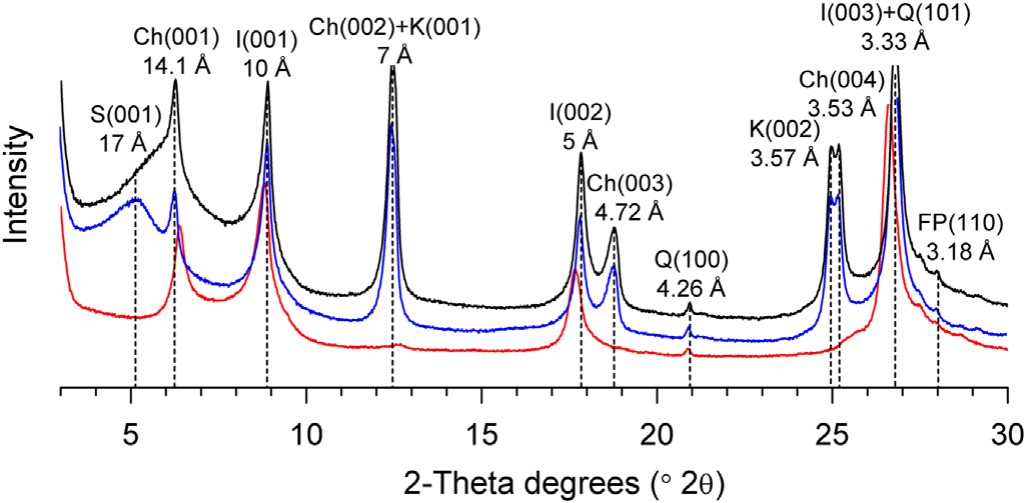
XRD patterns of clay fractions from the representative samples of the ZL red clay. Air dried (black curve), ethylene glycol (blue curve), heated (red curve). S-Smectite; Ch-Chlorite; K-Kaolinite; I-Illite; Q-Quarts; FP-Feldspar.

**Fig. 3:**
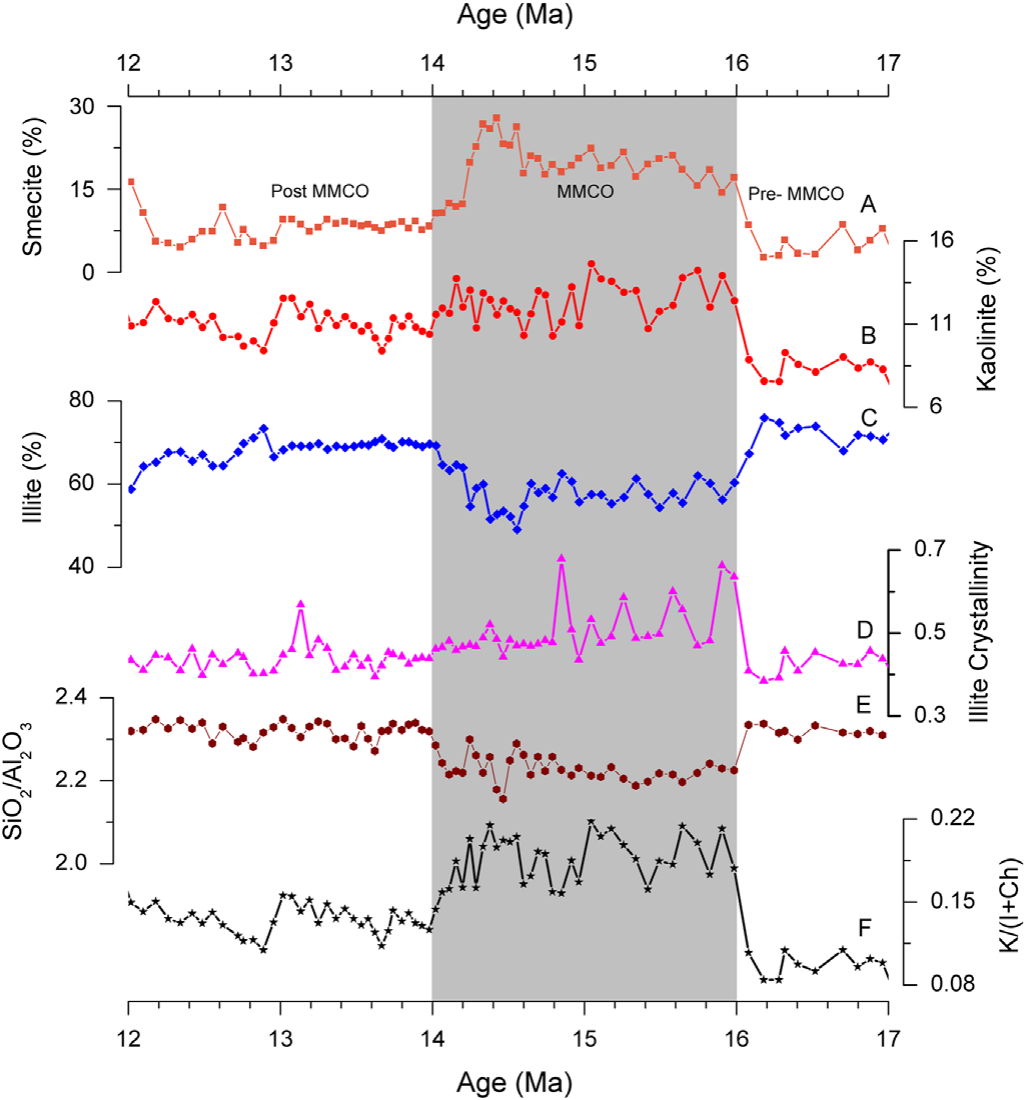
The Variations in clay minerals composition (%), illite crystallinity, SiO_2_/Al_2_O_3_ and K/(I+Ch) of the ZL red clay in the MMCO period. K/(I+Ch)-Kaolinite/(Illite+Chlorite).

Fig. 2 represents X-ray diffraction (XRD) patterns of clay fractions from the representative sample and Fig. 3 indicates the variations of clay minerals composition and characteristic indices of the ZL red clay in the MMCO period.

## 2 Experimental design, materials, and methods

### 2.1 Materials

The ZL drilling site (35°13′N, 106°05′E) is located at the eastern margin of the Longzhong Basin, near the Liupan Mts, which divides the Chinese Loess Plateau into two parts (Fig. 1). The ZL drilling core combing from two parallel drilling sites has a composite length of 654 m and detailed magnetostratigraphic correlations indicated that the ZL drilling core spanned from 25.6 to 4.8 Ma [2]. According to the magnetostratigraphy [2], grain size record [3], magnetic susceptibility variations [2], [4] of the ZL core, we chose 80 samples at depths from 240 m (12 Ma) to 398 m (17 Ma) for clay minerals and geochemical analysis [1].

### 2.2 X-ray diffraction measurements

The isolation of < 2 μm clay fractions was followed by the Chinese oil and gas industry standard analysis SY/T 5163-2010 [5]. The air-dry samples were treated with H_2_O_2_ to remove organic matter and dilute HCl to remove the calcium carbonate; the clay fractions were separated by gravity separation using Stoke's Law determinations and centrifugation in deionized water to remove free ions. In order to identify and quantify the clay minerals, XRD analysis of the oriented mounts was carried out for each sample under natural (air-dried) conditions (N), ethylene-glycol solvation for 24 h in a desiccator (EG), and heating at 550 °C for 2 h (H) [6], [7]. XRD patterns were obtained using a PANalytical X'Pert Pro MPD diffractometer with CuKα radiation and Ni filter, under a voltage of 40 kV and a current of 40 mA. The scans were performed from 3° to 30°, with a step size of 0.0167°. And a high resolution scan from 24° to 26° 2θ at a rate of 0.1° 2θ per minute was carried out to isolate the contributions of kaolinite and chlorite in the 3.5 Å peak. The ratio of kaolinite to chlorite was calculated from the peak areas of 3.58 Å and 3.53 Å. The identification of clay minerals was primarily based on the position of the (001) series of basal reflections in the three XRD patterns. The XRD experiment was made at the State Key Laboratory of Loess and Quaternary Geology, Institute of Earth Environment, Chinese Academy of Sciences.

Semi-quantitative analysis of clay minerals were determined from the basal reflection peak areas. Smectite (17 Å) (including random illite/smectite mixed-layers), illite (10 Å) and kaolinite+chlorite (7 Å) were performed on the ethylene-glycol curve by the XRD diagram using MacDiff 4.2.6 software [8], [9]. The relative abundances of clay minerals were determined using this formula: 4×I(illite 10 Å)+I(smectite 17 Å)+2×I(kaolinite, chlorite 7 Å) = 100%.

### 2.3 X-ray fluorescence measurements

Concentrations of major elements of clay fractions were measured by using a Philips PW4400 X-ray Fluorescence (XRF) spectrometer at the State Key Laboratory of Loess and Quaternary Geology, Institute of Earth Environment, Chinese Academy of Sciences. All samples were dried at low temperature (43 °C) for 72 h and ground to about < 75 μm (a 200 mesh size). About 0.6 g powder clay fraction samples were mixed with 6 g of Li_2_B_4_O7–Li_2_CO_3_ fusion reagent in Platinum crucibles; the mixed samples were placed in a Claisse Fluxy melting furnace and fused for 5 min at high temperature (about 1000 °C) before being formed into a glass sheet [10], [11]. The calibration curve was established using 16 Chinese National Standard soil reference samples (GSS-1 to GSS-16) [12], [13]. The reproducibility of elemental measurements were evaluated by repeat analysis using the National Standard soil reference sample GSS-8, with analytical uncertainties < 3% for major elements.
